# Supplementary material for: Causal Relationship between Aging and Anorexia Nervosa: A White-Matter-Microstructure-Mediated Mendelian Randomization Analysis
Source: Biomedicines. 2024 Aug 16;12(8):1874. doi: 10.3390/biomedicines12081874 (PMC11351342; doi:10.3390/biomedicines12081874)
Supplement: Supplementary file 1 [file biomedicines-12-01874-s001.zip › Supplementary Figure/Figure S8/Leaveoneout plot of MR analysis for TL to ubm-b-1799.pdf]

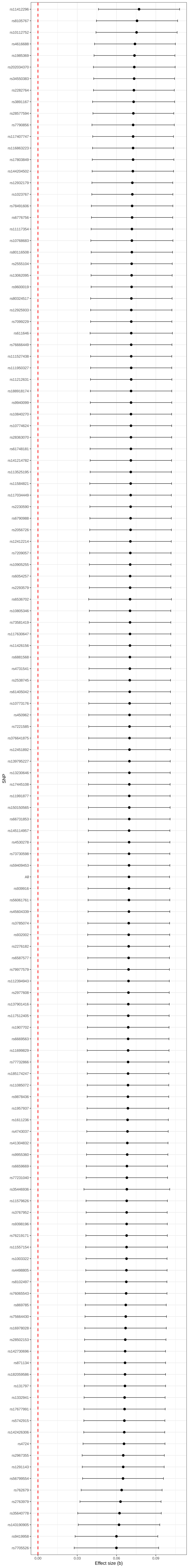

rs11412296
rs8105767
rs10112752
rs4616688
rs1985369
rs202034370
rs34550383
rs2282764
rs3891167
rs28577594
rs7790856
rs117407747
rs116863223
rs17803849
rs144204502
rs12932179
rs1023767
rs78491606
rs6776756
rs11117354
rs10768683
rs80116508
rs2555104
rs13062095
rs9600019
rs80324517
rs12925933
rs7099229
rs611646
rs76666449
rs111527438
rs111950327
rs11212631
rs188918174
rs9940099
rs10840270
rs10774624
rs28363070
rs61748181
rs141214782
rs113525195
rs11584821
rs117034449
rs2230590
rs6790988
rs2056726
rs12412214
rs7209057
rs10905255
rs6054257
rs2293579
rs6536702
rs10805346
rs73581419
rs117630647
rs11426156
rs6881568
rs4731541
rs2538745
rs61405042
rs10773176
rs450962
rs7221585
rs376641875
rs12451892
rs139795227
rs13230646
rs17445108
rs11991877
rs150150565
rs66731853
rs145114957
rs4530278
rs73730598
rs59409453
All
rs939916
rs56061761
rs45604339
rs3785074
rs932002
rs2276182
rs6587577
rs79977579
rs112394943
rs2977608
rs137901416
rs117512405
rs1907702
rs6669563
rs11699829
rs77732866
rs185174247
rs11085072
rs9878436
rs1957937
rs1611236
rs4743037
rs41304832
rs9955360
rs6659669
rs77231040
rs35446936
rs11579626
rs3767952
rs9398196
rs76219171
rs11557154
rs1003322
rs4498805
rs8102497
rs76065543
rs869785
rs75664430
rs16978028
rs28502153
rs142730696
rs871134
rs182059586
rs131797
rs1332941
rs17677991
rs5742915
rs142426306
rs4724
rs2967355
rs1291143
rs56799554
rs762679
rs2763979
rs35640778
rs143190905
rs9419958
rs7705526
SNP
0.00
0.03
0.06
0.09
Effect size (b)
